# Supplementary material for: Biological Compositions of Canine Amniotic Membrane and Its Extracts and the Investigation of Corneal Wound Healing Efficacy In Vitro
Source: Vet Sci. 2022 May 9;9(5):227. doi: 10.3390/vetsci9050227 (PMC9143045; doi:10.3390/vetsci9050227)
Supplement: Supplementary file 1 [file vetsci-09-00227-s001.zip › vetsci-1692932-supplementary.pdf]

Supplementary Materials

Figure S1. Relevant proteins detection in canine amniotic membrane and its extracts, uncropped full-length images of Western blot membranes.

HGF protein expression in cAM.

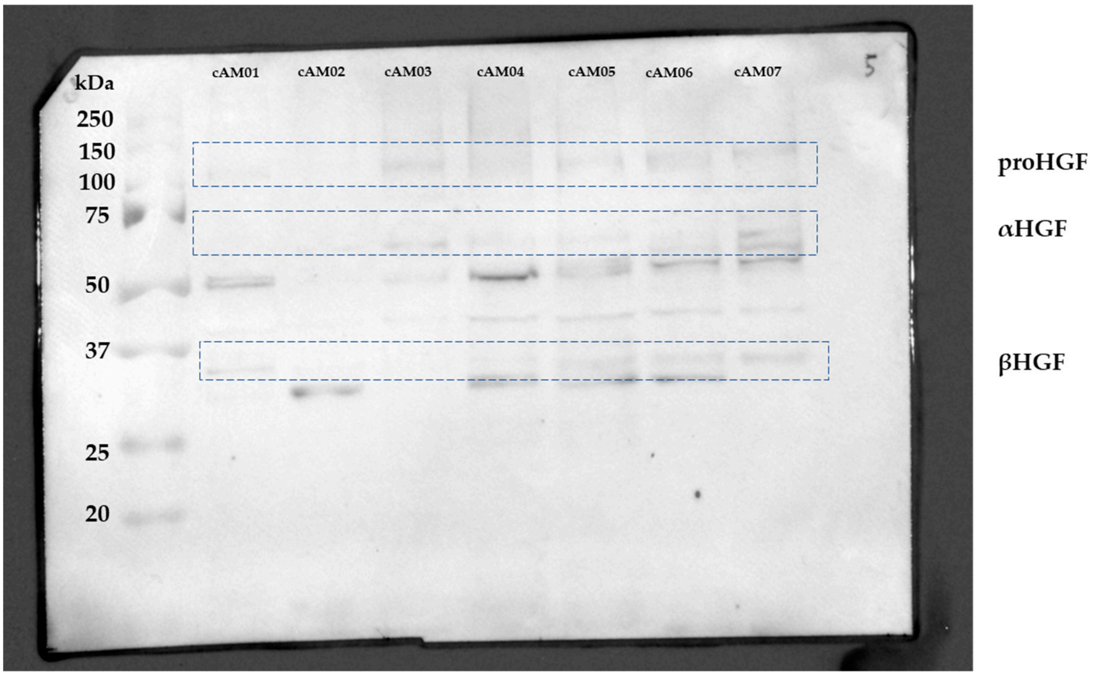

HGF protein expression in cAME.

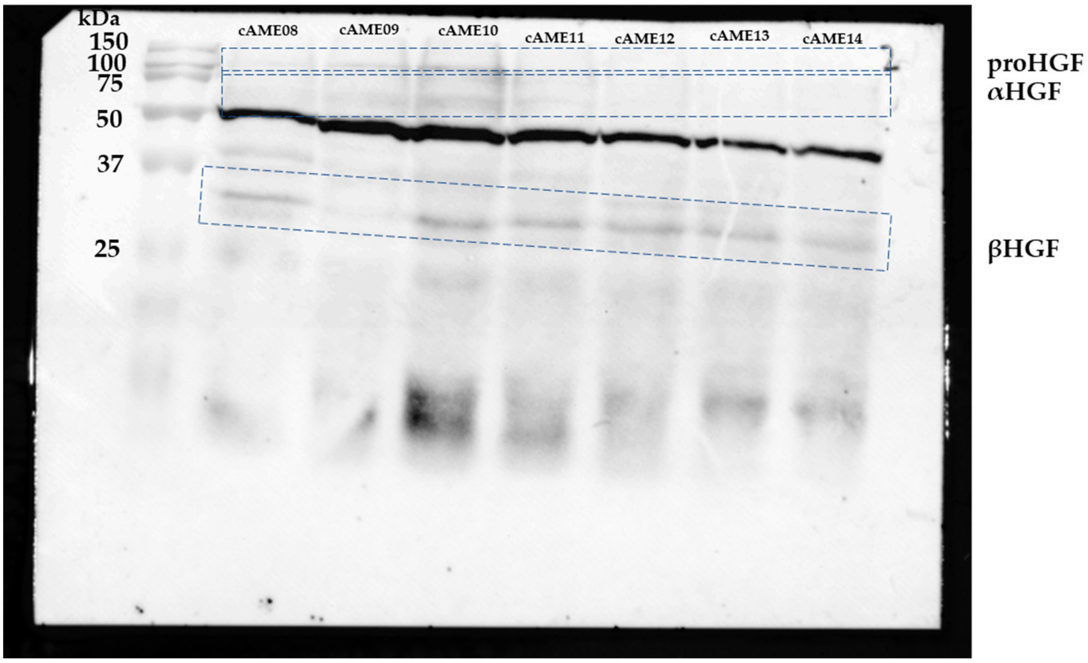

HGF protein expression in cAMX.

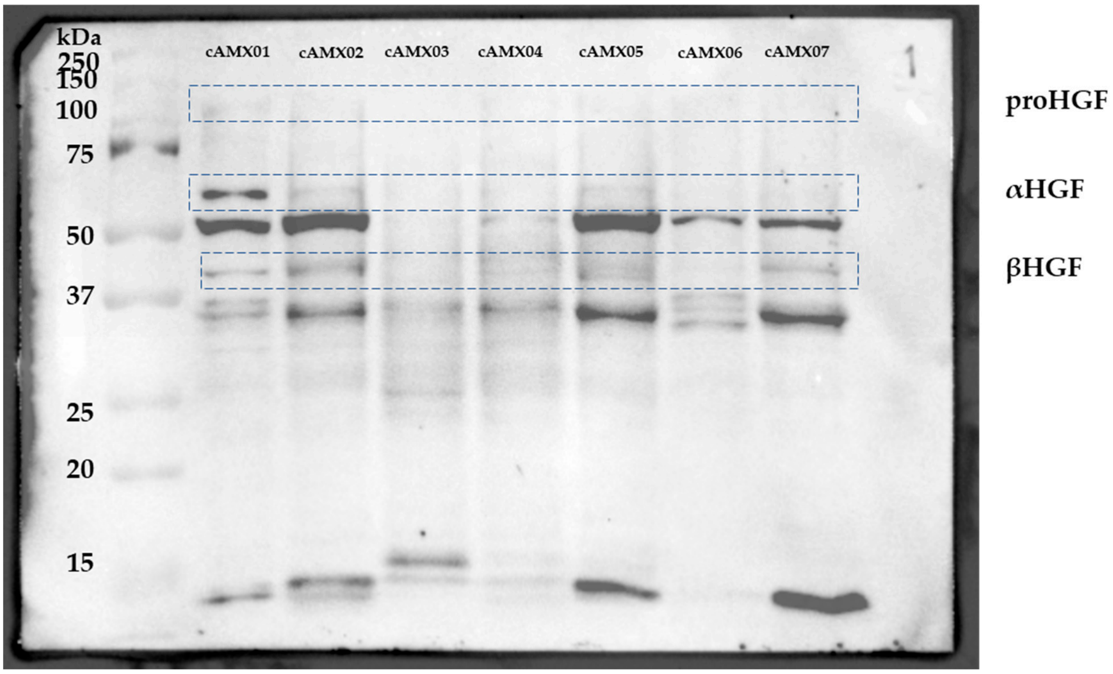

Western blot membranes of HGF (molecular weight of proHGF ~90 kDa, αHGF ~69 kDa and βHGF ~34 kDa). Proteins separated from SDS-PAGE were transferred onto a 0.2 μm PVDF membrane using a Trans-Blot Turbo Transfer System (Bio-Rad Laboratories Inc., Hercules, CA, USA). HGF protein was detected using HGF antibody (1:500, Thermo Fisher Scientific, Waltham, MA, USA) and anti-rabbit IgG HRP conjugated antibody (1:5000, R&D Systems Inc., Minneapolis, MN, USA) as a secondary antibody. Molecular weight (kDa) was determined by Bio-Rad Precision Plus Protein™ Dual Color Standards, 10 to 250 kDa; catalogue number: 1610374 (Bio-Rad Laboratories Inc., Hercules, CA, USA). Clarity™ Western ECL Substrate reagents (Bio-Rad Laboratories Inc., Hercules, CA, USA) were used for band detection and the ChemiDoc Imaging System (Bio-Rad Laboratories Inc., Hercules, CA, USA) was used for visualization. Band intensities of HGF protein were normalized with total protein intensities (Pierce™ Reversible Protein Stain Kit for PVDF Membranes, Thermo Fisher Scientific, Waltham, MA, USA) and analyzed using Bio-Rad Image Lab Software (version 6.0.1, Bio-Rad Laboratories Inc., Hercules, CA, USA). Each lane represented each sample in each group.

TIMP-1 protein expression in cAM.

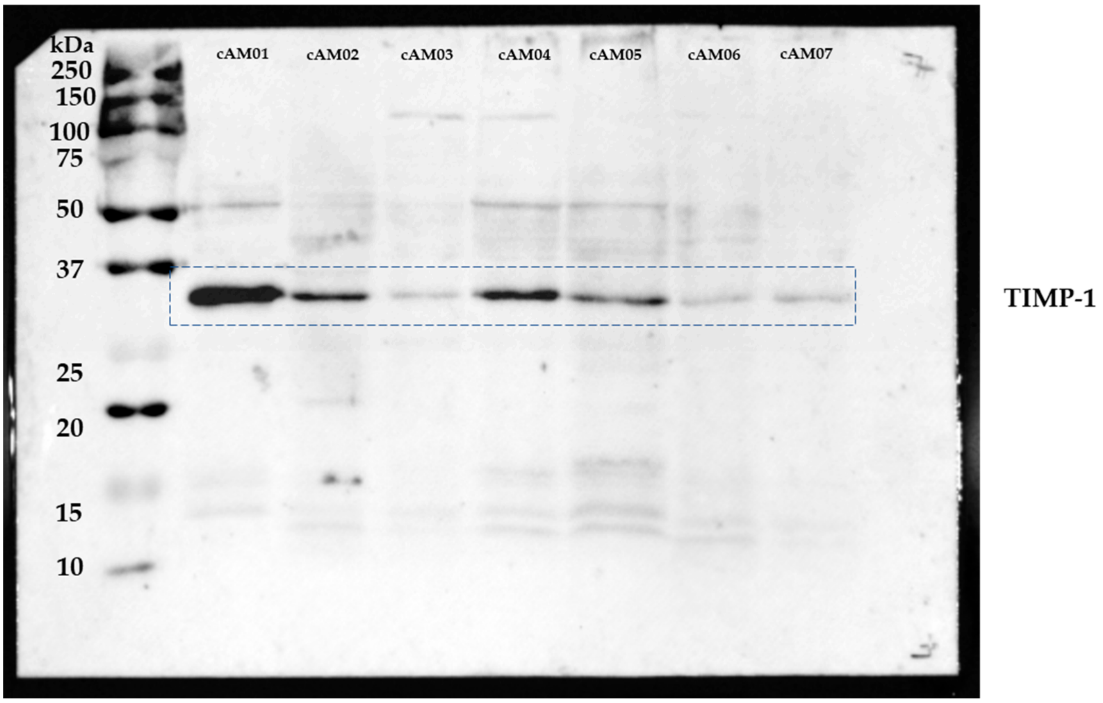

TIMP-1 protein expression in cAME.

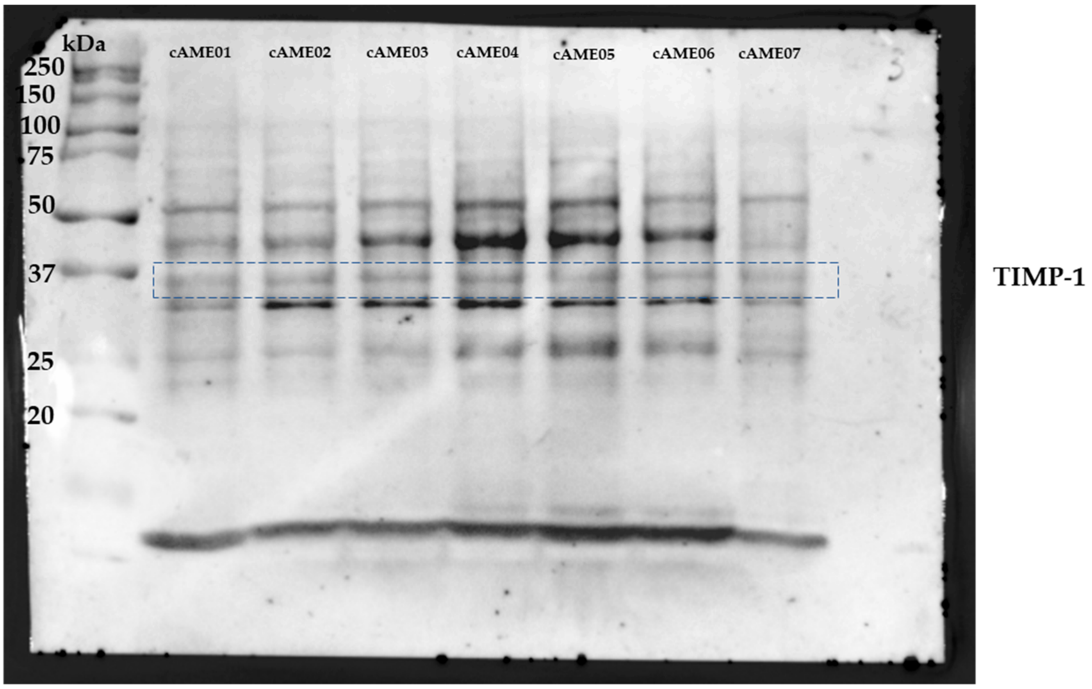

TIMP-1 protein expression in cAMX.

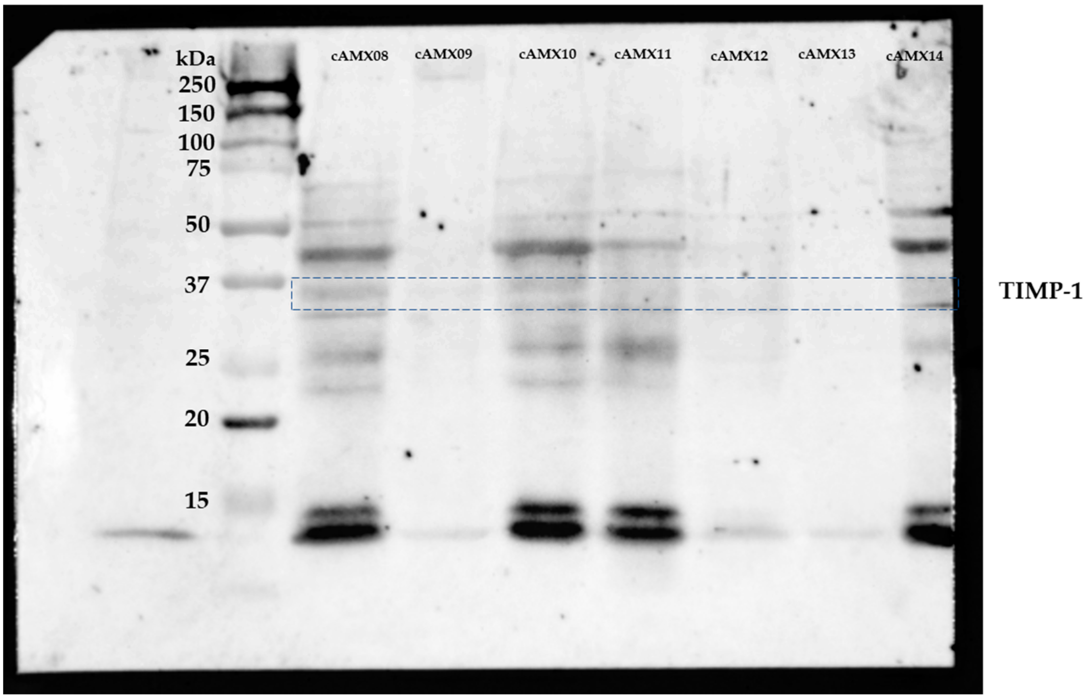

Western blot membranes of TIMP-1 (molecular weight of TIMP-1 ~35 kDa). Proteins separated from SDS-PAGE were transferred onto a 0.2 μm PVDF membrane using a Trans-Blot Turbo Transfer System (Bio-Rad Laboratories Inc., Hercules, CA, USA). TIMP-1 protein was detected using TIMP-1 antibody (1:1000, Thermo Fisher Scientific, Waltham, MA, USA) and m-IgGk BP-HRP (1:10,000, Santa Cruz Biotechnology, Dallas, TX, USA) as a secondary antibody. Bio-Rad Precision Plus Protein™ Dual Color Standards, 10 to 250 kDa; catalogue number: 1610374 (Bio-Rad Laboratories Inc., Hercules, CA, USA) was used as molecular weight marker (kDa). Clarity™ Western ECL Substrate (Bio-Rad Laboratories Inc., Hercules, CA, USA) was used for band detection and the ChemiDoc Imaging System (Bio-Rad Laboratories Inc., Hercules, CA, USA) was used for visualization. Band intensities of TIMP-1 protein were normalized with total protein intensities (Pierce™ Reversible Protein Stain Kit for PVDF Membranes, Thermo Fisher Scientific, Waltham, MA, USA) and analyzed using Bio-Rad Image Lab Software (version 6.0.1, Bio-Rad Laboratories Inc., Hercules, CA, USA). Each lane represented each sample in each group.

TIMP-2 protein expression in cAM.

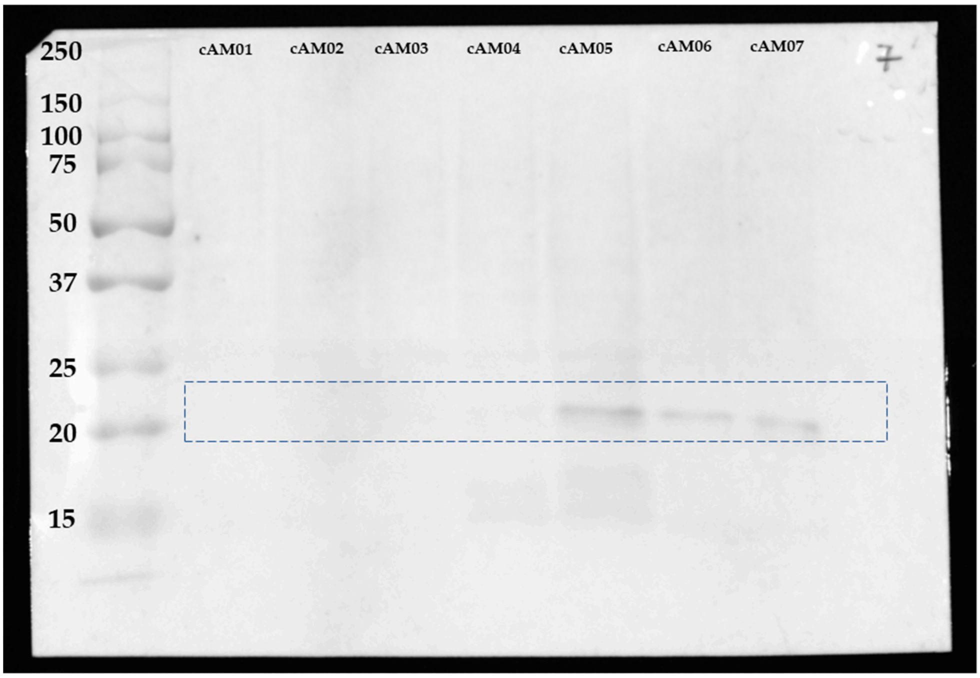

TIMP-2 protein expression in cAME.

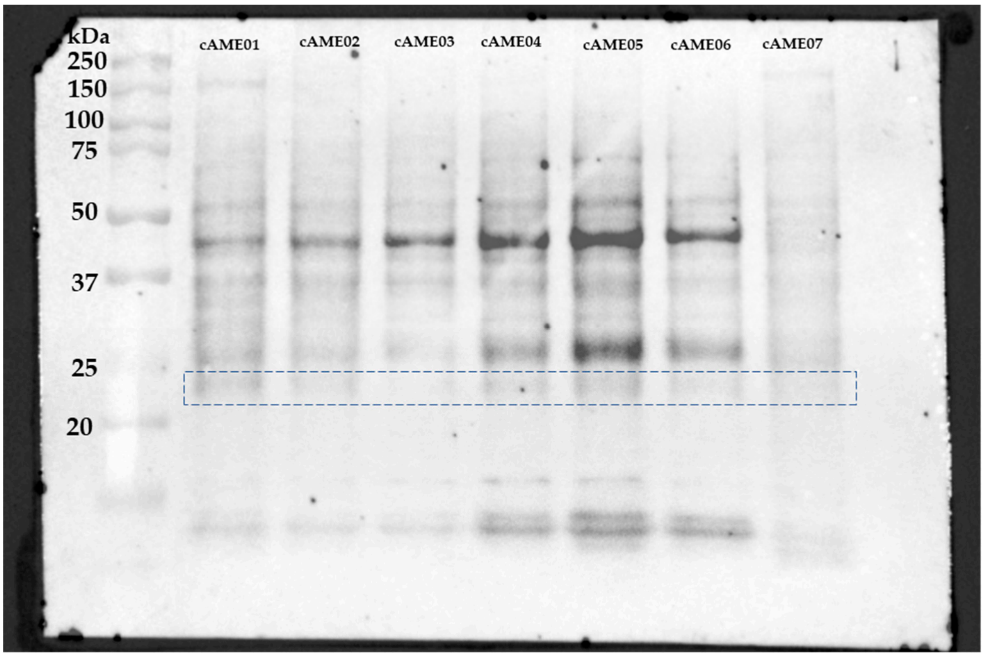

TIMP-2 protein expression in cAMX.

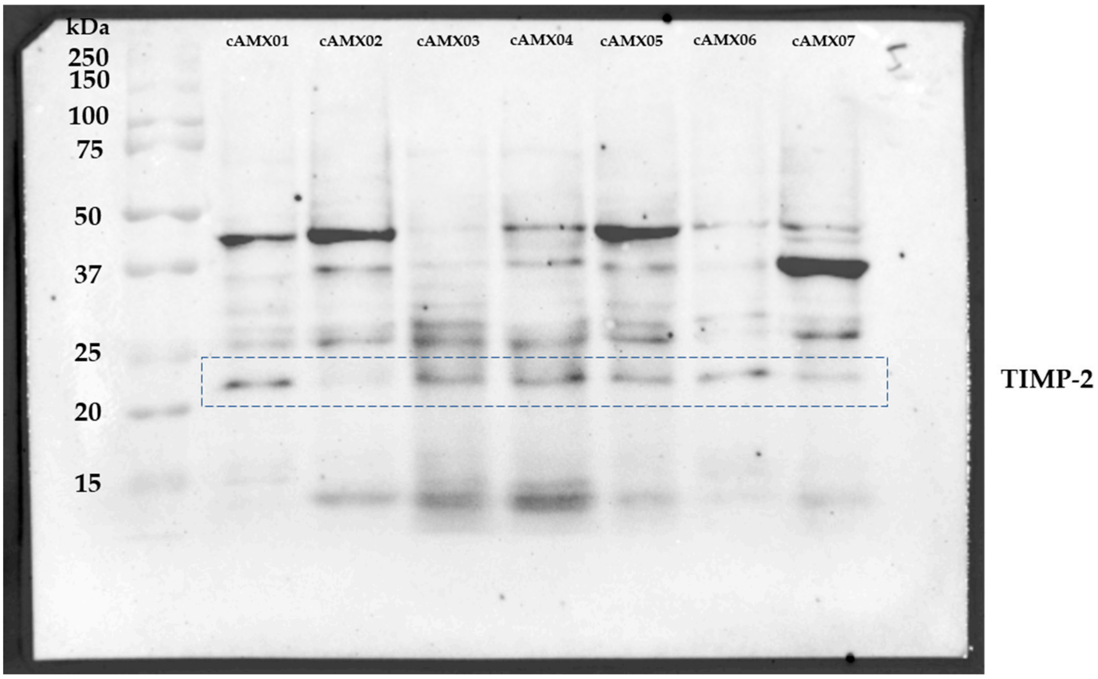

Western blot membranes of TIMP-2 (molecular weight of TIMP-2 ~22 kDa). Proteins separated from SDS-PAGE were transferred onto a 0.2 μm PVDF membrane using a Trans-Blot Turbo Transfer System (Bio-Rad Laboratories Inc., Hercules, CA, USA). TIMP-2 protein was detected using TIMP-2 antibody (1:1000, Thermo Fisher Scientific, Waltham, MA, USA) and m-IgGk BP-HRP (1:10,000, Santa Cruz Biotechnology, Dallas, TX, USA) as a secondary antibody. Molecular weight marker (kDa): Bio-Rad Precision Plus Protein™ Dual Color Standards, 10 to 250 kDa; catalogue number: 1610374 (Bio-Rad Laboratories Inc., Hercules, CA, USA). Band detection was done by Clarity™ Western ECL Substrate (Bio-Rad Laboratories Inc., Hercules, CA, USA) and visualization using ChemiDoc Imaging System (Bio-Rad Laboratories Inc., Hercules, CA, USA). Band intensities of TIMP-2 protein were normalized with total protein intensities (Pierce™ Reversible Protein Stain Kit for PVDF Membranes, Thermo Fisher Scientific, Waltham, MA, USA) and analyzed using Bio-Rad Image Lab Software (version 6.0.1, Bio-Rad Laboratories Inc., Hercules, CA, USA). Each lane represented each sample in each group.

TSP-1 protein expression in cAM.

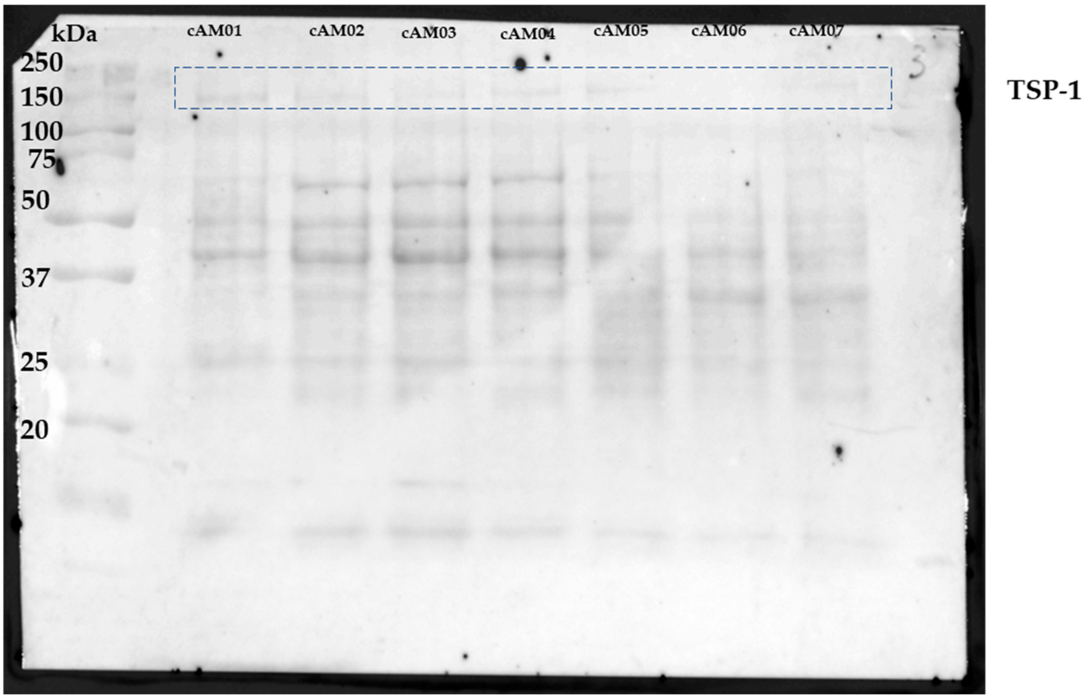

TSP-1 protein expression in cAME.

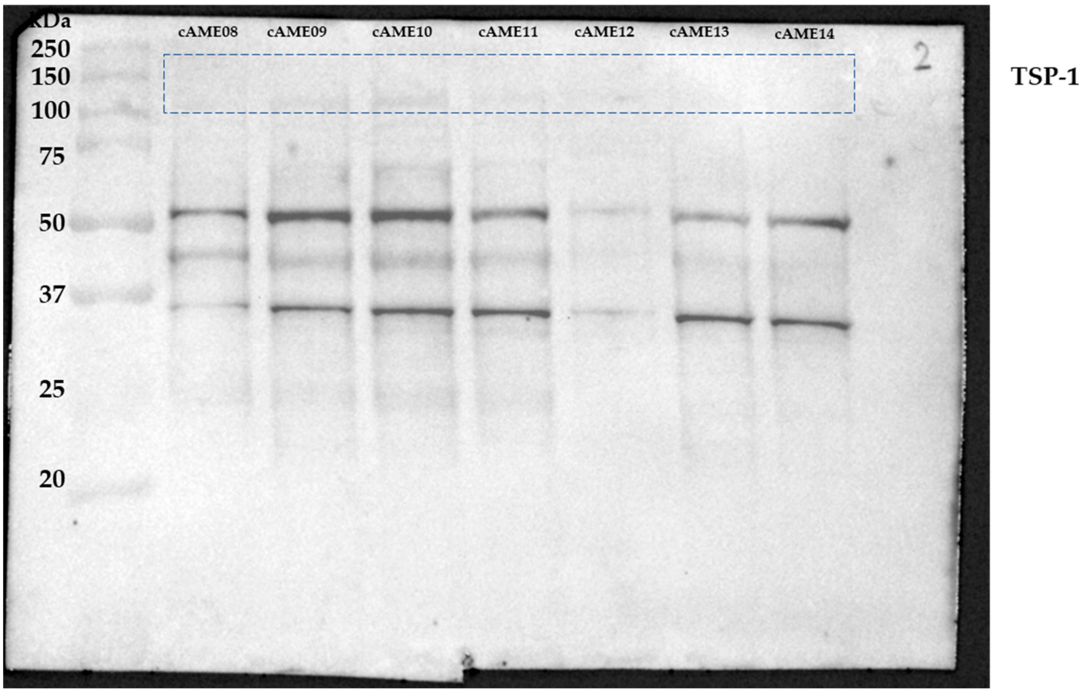

TSP-1 protein expression in cAMX.

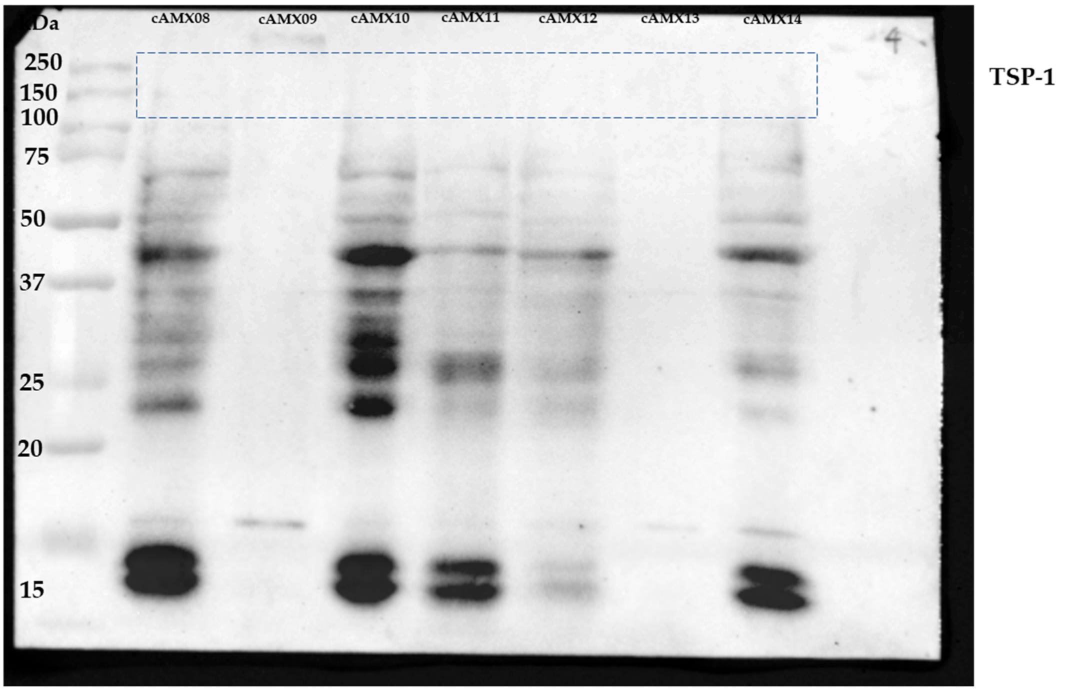

Western blot membranes of TSP-1 (molecular weight of TSP-1 ~169-198 kDa). Proteins separated from SDS-PAGE were transferred onto a 0.2 μm PVDF membrane using a Trans-Blot Turbo Transfer System (Bio-Rad Laboratories Inc., Hercules, CA, USA). Protein was detected using TSP-1 antibody (1:500, Santa Cruz Biotechnology, Dallas, TX, USA) and m-IgGk BP-HRP (1:10,000, Santa Cruz Biotechnology, Dallas, TX, USA) as a secondary antibody. For molecular weight (kDa) determination, Bio-Rad Precision Plus Protein™ Dual Color Standards, 10 to 250 kDa; catalogue number: 1610374 (Bio-Rad Laboratories Inc., Hercules, CA, USA) was used. Band detection was done by using Clarity™ Western ECL Substrate (Bio-Rad Laboratories Inc., Hercules, CA, USA) and visualization using ChemiDoc Imaging System (Bio-Rad Laboratories Inc., Hercules, CA, USA). Band intensities of TSP-1 protein were normalized with total protein intensities (Pierce™ Reversible Protein Stain Kit for PVDF Membranes, Thermo Fisher Scientific, Waltham, MA, USA) and analyzed using Bio-Rad Image Lab Software (version 6.0.1, Bio-Rad Laboratories Inc., Hercules, CA, USA). Each lane represented each sample in each group.

IL-1RA protein expression in cAM.

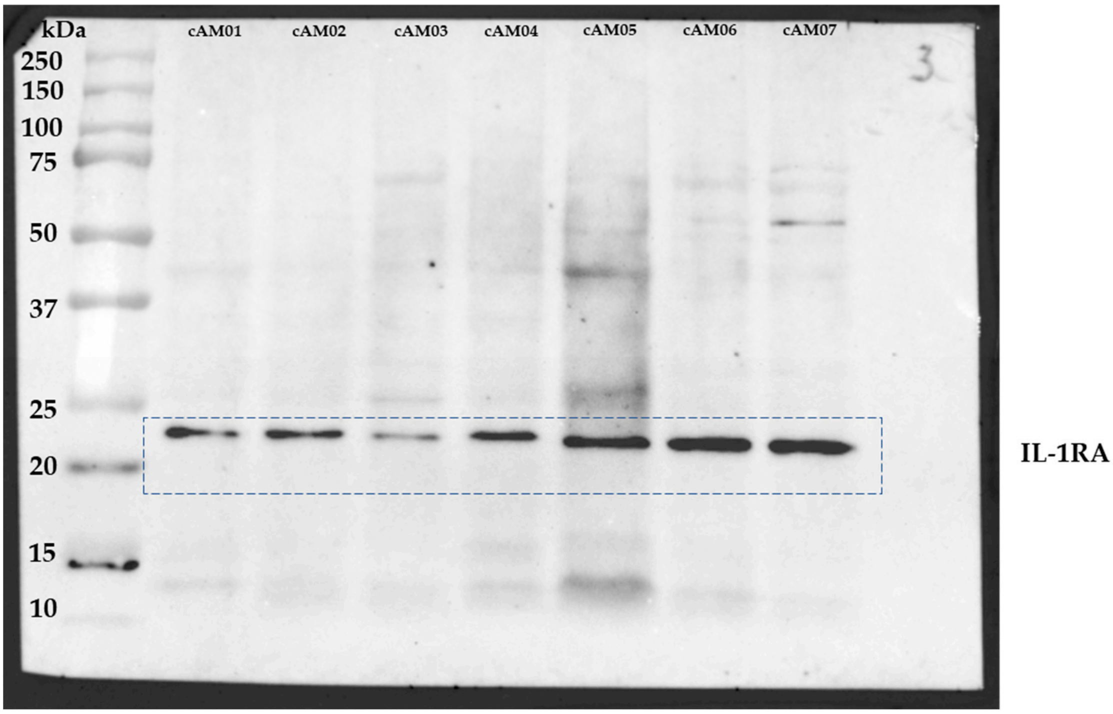

IL-1RA protein expression in cAME.

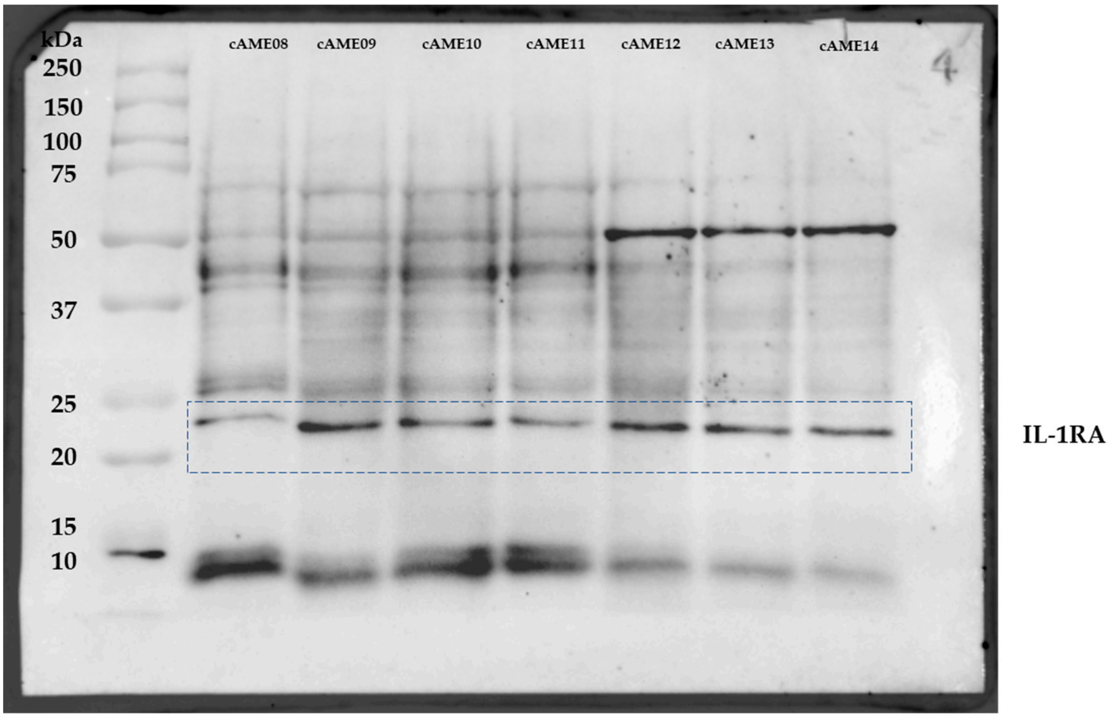

IL-1RA protein expression in cAMX.

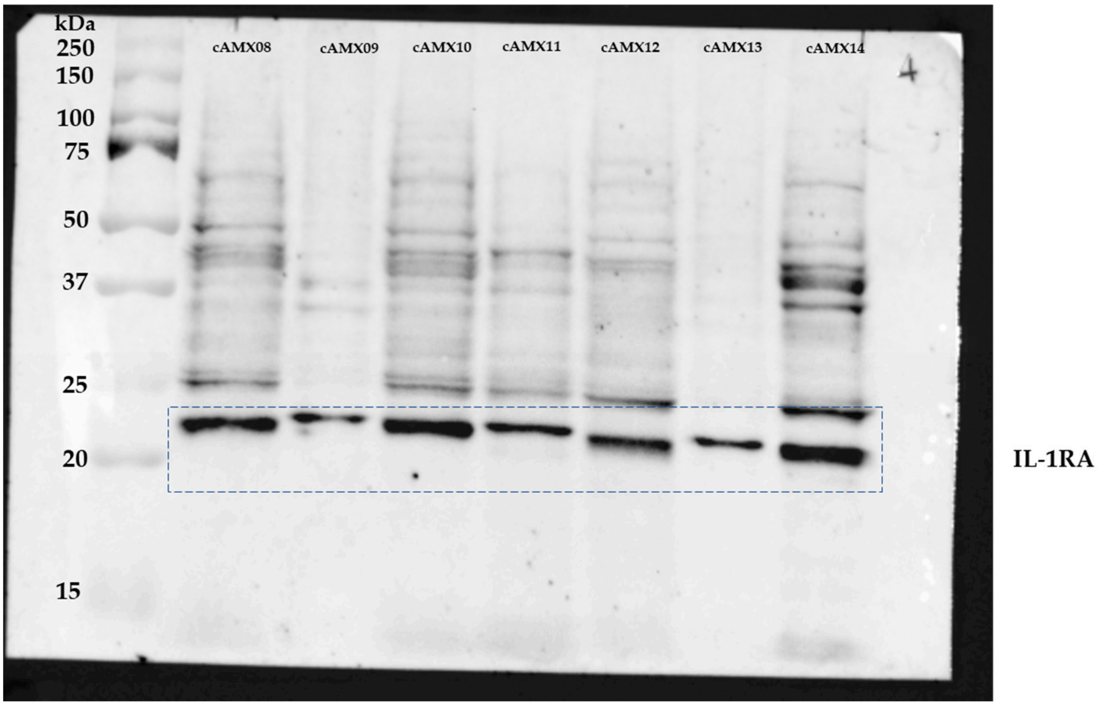

Western blot membranes of IL-1RA (molecular weight of IL-1RA ~18-22 kDa). Proteins separated from SDS-PAGE were transferred onto a 0.2 μm PVDF membrane using a Trans-Blot Turbo Transfer System (Bio-Rad Laboratories Inc., Hercules, CA, USA). IL-1RA protein was detected using IL-1RA antibody (1:500, Thermo Fisher Scientific, Waltham, MA, USA) and anti-rabbit IgG HRP conjugated antibody (1:5000, R&D Systems Inc., Minneapolis, MN, USA) as a secondary antibody. To determine molecular weight (kDa), Bio-Rad Precision Plus Protein™ Dual Color Standards, 10 to 250 kDa; catalogue number: 1610374 (Bio-Rad Laboratories Inc., Hercules, CA, USA) was used. Clarity™ Western ECL Substrate (Bio-Rad Laboratories Inc., Hercules, CA, USA) was used for band detection and the ChemiDoc Imaging System (Bio-Rad Laboratories Inc., Hercules, CA, USA) was used for visualization. Band intensities of IL-1RA protein were normalized with total protein intensities (Pierce™ Reversible Protein Stain Kit for PVDF Membranes, Thermo Fisher Scientific, Waltham, MA, USA) and analyzed using Bio-Rad Image Lab Software (version 6.0.1, Bio-Rad Laboratories Inc., Hercules, CA, USA). Each lane represented each sample in each group.
